# Preparation of Triple-Functionalized Montmorillonite Layers Promoting Thermal Stability of Polystyrene

**DOI:** 10.3390/nano11092170

**Published:** 2021-08-25

**Authors:** Chengcheng Yu, Xu Hu, Shichao Lu, Yangchuan Ke, Jianbin Luo

**Affiliations:** 1 State Key Laboratory of Tribology, Department of Mechanical Engineering, Tsinghua University, Beijing 100084, China; luojb@tsinghua.edu.cn; 2 CNPC Nanochemistry Key Laboratory, College of Science, China University of Petroleum, Beijing 102249, China; xuhu0001@126.com (X.H.); shichaolu@yeah.net (S.L.); kyc010@sohu.com (Y.K.)

**Keywords:** montmorillonite functionalization, acid treatment, polymer–clay nanocomposite, thermal properties, polystyrene

## Abstract

The objective of this study was to investigate the effect of three different treatments on the morphology, microstructure, and the thermal characteristics of a montmorillonite (Mt) sample, by using hydrochloric acid (HCl), tributyl tetradecyl phosphonium chloride (TTPC) surfactant, and γ-methacryloxypropyltrimethoxysilane (γ-MPS). The resultant nanofillers were characterized by Fourier transform infrared (FTIR), X-ray diffraction (XRD), transmission electron microscopy (TEM), nitrogen adsorption–desorption analysis, X-ray fluorescence spectrometry (XRF), and thermogravimetric analysis (TGA). The results showed that the amount of chemical grafting of the γ-MPS was increased after the acid treatment, whereas the amount of intercalation of the TTPC surfactant was decreased. The preintercalation of TTPC or silylation of γ-MPS, for the Mt sample, had a certain hindrance effect on its subsequent silylation or intercalation treatments. Furthermore, the effect of four different nanofillers on the thermal stability properties of the polystyrene (PS) matrix were also investigated. The results showed an increase in thermal stability for the triple-functionalized Mt, compared with the double-functionalized samples. The onset decomposition temperatures and the maximum mass loss temperatures of the PS nanocomposites were increased by 27 °C and 32 °C, respectively, by the incorporation of triple-modified Mt, as a result of the good exfoliation and dispersion of the nanolayers, more favorable polymer–nanofiller interaction, as well as the formation of a more remarkable tortuous pathway in the continuous matrix.

## 1. Introduction

Polymer/clay nanocomposites have attracted much attention for various applications, due to their unprecedented improvement in their performance properties compared to virgin polymers or traditional micro- and macro-composites [1,2]. Important improvements in the mechanical, barrier, thermal, and fire retardant properties were obtained in the nanocomposites that were prepared with organoclays [3,4]. The final properties of nanocomposites depend on several factors, such as the properties of each component, contents, dimensions, orientations of the clay, and the interfacial interactions between the matrix and the clay layers [5]. Among them, the interfacial interactions are mainly determined by the chemical compatibility between the polymer matrix and the layered silicates. Among the layered silicates, montmorillonite (Mt) and bentonite are two important clays, and much attention has been paid to Mt to prepare organoclays, due to its excellent properties, such as a high cation exchange capacity, large surface area, and strong adsorption capacity [6]. The surfaces of the Mt layers can have a deficit of positive charges because of the isomorphic substitution, which are neutralized by the adsorption of hydrated cations, such as Na^+^ or Ca^2+^, inside the interlayer spacing and on the surface of the clay platelets [7,8]. Thus, the Mt layers are inherently hydrophilic and incompatible with the vast majority of polymers. However, it is known that the homogeneous dispersion of silicate layers, throughout the polymer matrix, is usually desirable for maximum reinforcement properties, compared with the polymer matrix [9]. Therefore, to increase the interfacial interaction and chemical compatibility between the two phases, the hydrophilic Mt surface must be modified to an organophilic surface. The organic treatment of the surface not only increases the interlayer spacing between the silicate layers, but also improves the compatibility between the clay and polymer matrix, which may facilitate the intercalation and exfoliation of clay layers inside the bulk hydrophobic polymers, and thus offers reinforcing effects [10].

The modification of Mt, with various methods, has been investigated previously [11,12,13]. The conventional and straightforward method that is used to prepare organophilic Mt is the cation exchange reaction with quaternary ammonium surfactants, which are intercalated into the interlayer spacing and replace the exchangeable cations [14]. However, the alkyl ammonium surfactants that are used in commercially modified Mt production decompose at temperatures above 170–180 °C, and thus are not suitable for polymers that are processed at higher temperatures [15]. Therefore, to overcome this difficulty, the functionalization of Mt with phosphonium surfactants has attracted great interest in the last few years [16]. Alkyl phosphonium surfactants are typical intercalating agents for the preparation of highly thermally stable organoclays, compared with ammonium surfactants [17]. The intercalation of phosphonium surfactants into the interlayer of Mt increases the interlayer spacing and decreases the surface energy, which results in the improvement of the compatibility with hydrophobic polymers [18].

The activation treatment of clay samples with inorganic acids is commonly used for the production of adsorbents or catalysts that are used in environmental protection. The edges of the crystals are opened, and cations (Al^3+^, Mg^2+^) in the octahedral sheets are exposed to acid and become soluble, and the external specific surface area and pore diameter are increased, due to the dissolution of structural ions [19]. Moreover, the acid treatment of Mt can dissolve impurities and replace the exchangeable cations with hydrated protons [20]. In general, the acid treatment of clay is used before the organic modification reactions.

The electrostatic interactions between the surfactants and the clay layers do not provide an efficient linkage between the clay surface and polymer matrix. Therefore, the chemical functionalization of Mt with a silane coupling agent has attracted great attention in recent years [21]. The silylation reaction, also known as silane grafting, between the silane molecules and reactive silanol groups, located at the broken edges and at the structural defects situated at the interlayer spacing and external surface of the clay layers, is proven to be an efficient method to modify the clay surfaces [22]. The reaction of silylation is an irreversible process, because chemical bonds, with a covalent character, are formed between the silicates and organic components [23]. The silylation reaction on the internal surface of clay can cause an increase in the interlayer spacing, while the silylation taking place on the external surfaces and edges brings about a less conspicuous change, or no change, in the interlayer spacing [24]. In addition, the dispersion of the modified clay samples in the polymer matrix is improved when polymer chains are attached to the clay surfaces by covalent bonds.

Polystyrene (PS) is a synthetic thermoplastic that is extensively used in various applications, due to its outstanding properties, such as high abrasion resistance, strong load bearing capacity, and convenience of processing. However, its relatively weak thermal stability greatly limits the applications in a variety of areas, such as lubricating materials in oil and gas drilling engineering. To overcome this drawback, the inorganic layered silicates have been used to enhance the thermal properties of the virgin PS [25].

To our knowledge, only a few reports described the effect of one method on the further modification by other two methods, for the alteration of Mt [26]. In this article, the triple-functionalized Mt was prepared successfully by using combined reactions of acid activation, phosphonium cation exchange, and polymerization active silane coupling agent grafting reaction. The effect of one treatment strategy on the subsequent modification by other two treatments has been investigated and compared. Furthermore, the significant role of modified Mt on the thermal stability of the polystyrene nanocomposites was extensively discussed.

## 2. Materials and Methods

### 2.1. Materials

Sodium montmorillonite (Mt), with a cationic exchange capacity (CEC) of 1 mmol/1 g of clay, was provided by the Huai An Saibei Technology Co. Ltd., Zhangjiakou, China. Hydrochloric acid (HCl, 37% *w*/*w*) was purchased from Beijing Chemical Reagents Company, Beijing, China. The tributyl tetradecyl phosphonium chloride (TTPC, 97%) and γ-methacryloxypropyltrimethoxysilane (γ-MPS, 97%) were purchased from Aladdin Industrial Corporation, Shanghai, China. Styrene (St, >98%) monomer and divinylbenzene (DVB, >80%) were supplied by the Tianjin Guang fu Fine Chemicals Research Institute, Tianjing, China. All the other chemicals were of analytical grade and were used without further purification. Deionized water was used in all experiments.

### 2.2. Acid Activation Reaction

The modified Mt with a mineral acid was synthesized via a previously reported method [27]. A total of 5.0 g of Mt was dispersed in 170 mL of deionized water and stirred for 30 min. Then, 30 mL of a 37% (*w*/*w*) HCl solution was added, and the mixture was mechanically stirred for 24 h at room temperature. The acid-activated clay was separated by centrifugation at 5000 rpm and washed with deionized water. The procedure was repeated several times until the pH of the solution was equal to 6. The resulting product was freeze-dried using a VIRTIS 2KBTES-55 freeze dryer and abbreviated as H-Mt.

### 2.3. Ion Exchange Reaction

The ion exchange reaction of clay was performed through the method reported in a previous paper [28]. In summary, a sample of clay (2.5 g) was dispersed in 75 mL of deionized water, and the dispersion was stirred vigorously for 30 min at 75 °C. Then, a solution of TTPC in 25 mL of deionized water was slowly added with stirring for 11 h, and the amount of TTPC was equivalent to 1.2 CEC of Mt. The mixture was filtered followed by washing several times with deionized water until no more trace of TTPC was detected by silver nitrate test. The product was named P-Mt. 

### 2.4. Silylation Reaction

**Figure 1:**
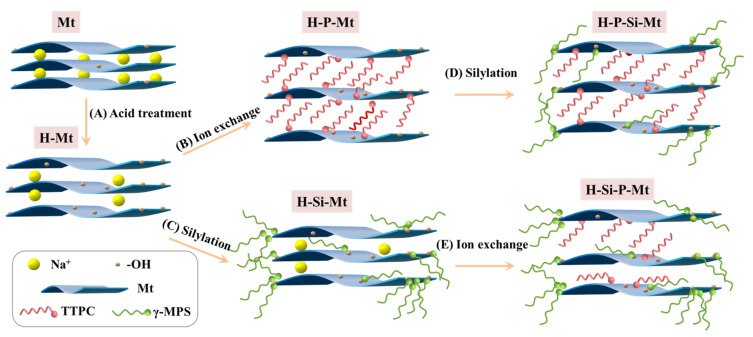
Schematic representation of the acid activation, ion exchange and silylation reactions of Mt samples. (**A**) The acid activation of Mt was performed under the treatment of HCl; (**B**) the TTPC surfactants were intercalated into the interlayer spacing of H-Mt; (**C**) the γ-MPS molecules were grafted onto the exterior surfaces and / or the broken edges of the H-Mt; (**D**) the H-P-Mt was modified with γ-MPS molecules to obtain the H-P-Si-Mt; **(E)** the H-Si-Mt was intercalated with TTPC surfactants to obtain the H-Si-P-Mt sample.

The silylation followed the procedure described in a previous report [29]. Then, 2.483 g of γ-MPS was dissolved in 100 mL of a methanol/deionized water solution (*v*/*v* = 9:1), and the pH was adjusted to about 4.0 with acetic acid with stirring for 1 h at room temperature. Then, a 2.5 g sample of clay was added, and the suspension was refluxed at 75 °C for 24 h. Finally, the resulting product was collected by filtration followed by washing four times with methanol and was referred to as Si-Mt. A schematic representation of the acid activation, ion exchange and silylation of Mt is shown in Figure 1.

### 2.5. Synthesis of The Polystyrene (PS)/Organic Mt (OMt) Nanocomposites

PS/OMt nanocomposites with OMt content of 3.0 wt.% (based on the weight of St) were prepared by the soap-free emulsion polymerization according our previous report [25]. In brief, a mixture of OMt, DVB and St was mechanically stirred for 1 h at room temperature. Then, the mixture was added into the deionized water with stirring in 500-mL three-neck round-bottomed flask under nitrogen atmosphere. When the temperature increased up to 75 °C, KPS (potassium persulfate) initiator was added, and the resulting mixture polymerized for 9 h to obtain the PS/OMt nanocomposites. Four different PS nanocomposites were prepared, and pure PS was also synthesized under the same conditions for comparison.

### 2.6. Characterization

Fourier transform infrared (FTIR) spectra were measured using an FTS-3000 spectrophotometer (Digilab Co. Ltd., MA, USA) within the range of 4000–400 cm^−1^ with a resolution of 4 cm^−1^ using the KBr pressed disk technique. X-ray diffraction (XRD) measurements were carried out with a Bruker D8 Advance X-ray diffractometer (Stadi P, Karlsruhe, Germany) with Cu Ka radiation (λ = 0.1540 nm) over a range of 2θ = 1.5 to 12° at a scan rate of 2°/min. The basal spacing reflection of the sample was computed by applying the Bragg’s law, and the voltage and current of the X-ray tubes were 40 kV and 30 mA, respectively. The morphology of the sample was determined by transmission electron microscopy (TEM, JEM-2100, JEOL Ltd., Tokyo, Japan) at an accelerating voltage of 200 kV. The samples were diluted with ethanol and then dispersions were placed onto a copper TEM grid and analyzed. The nitrogen adsorption and desorption analysis was performed using a Micromeritics ASAP 2420 instrument (Micromeritics, GA, USA). The Brunauer–Emmett–Teller (BET) surface areas and the Barrett–Joyner–Halenda (BJH) pore volumes of the clay samples were calculated for further confirmation of modified reaction. The chemical compositions of samples were determined by X-ray fluorescence spectrometry (XRF, Axios ^max^, PANalytical, the Netherlands). Elements lighter than oxygen could not be detected. Thermogravimetric analysis (TGA) was carried out on a thermogravimetric analyzer (TGA, STA409PC, Bavaria, Germany) at a heating rate of 10 °C/min under a nitrogen atmosphere between 30 and 800 °C. The differential thermogravimetry (DTG) curves were obtained from the data of TGA.

## 3. Results

In this section, the obtained sample, by the H-Mt, further modified with a cationic exchange of TTPC, as shown in Figure 1B, was named H-P-Mt. The sample that was prepared by HCl and then silylated with γ-MPS was labeled as H-Si-Mt (Figure 1C). Similarly, the Mt was modified, in turn, with HCl, TTPC, and γ-MPS, to obtain the triple-modified H-P-Si-Mt sample (Figure 1D). The H-Si-P-Mt sample was prepared by the acid activation, silylation, and ion exchange of Mt (Figure 1E). The P-Si-Mt and Si-P-Mt samples were also prepared and investigated.

### 3.1. FTIR Analysis of Modified Mt

**Figure 2:**
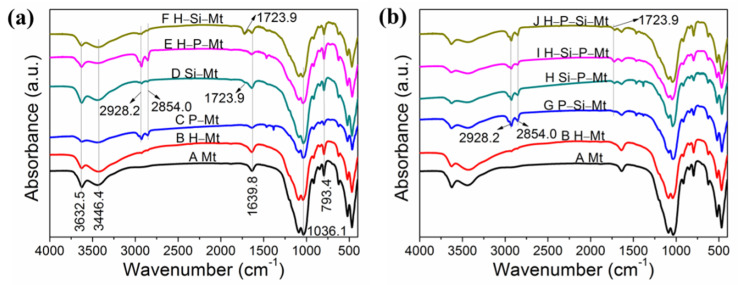
FTIR spectra obtained from the Mt and functionalized Mt samples. (**a**) FTIR spectra of (A) Mt, (B) H-Mt, (C) P-Mt, (D) Si-Mt, (E) H-P-Mt, and (F) H-Si-Mt; (**b**) FTIR spectra of (A) Mt, (B) H-Mt, (G) P-Si-Mt, (H) Si-P-Mt, (I) H-Si-P-Mt, and (J) H-P-Si-Mt.

The representative FTIR spectra of the Mt and modified Mt are shown in Figure 2. For the Mt, the broad absorption bands, at 3446.4 and 1639.8 cm^−1^, corresponded to the –OH stretching vibration and H–O–H bending vibration of free adsorbed water within the Mt interlayer, respectively. The characteristic absorption peak at 3632.5 cm^−1^ was attributed to the stretching vibration of the Al–OH and Si–OH groups, and these hydroxyl groups were responsible for the reaction with the silane coupling agent [30]. The bands at 1036.1 and 793.4 cm^−1^ were representative of the stretching vibration peaks of Si–O and Si–O–Al, respectively. In the case of H-Mt, the decrease in the intensity of the peak at 1036.1 and 793.4 cm^−1^ was due to changes in the Si environment produced by the acid treatment, suggesting that the textural character of Mt had been changed. Meanwhile, an increase in the intensity of the bands at 3632.5, 3446.4, and 1639.8 cm^−1^ was observed, indicating an increase in the amount of hydroxyl groups and adsorbed water, resulting from the interlayer Na^+^ was partly exchanged with protons during the acid treatment. The spectrum of Si-Mt not only had the characteristic bands of Mt, but also exhibited two new peaks at 2928.2 and 2854.0 cm^−1^, which were assigned to the antisymmetric and symmetric stretching vibrations of the –CH_2_ groups, respectively. In addition, the characteristic peak of γ-MPS, observed at 1723.9 cm^−1^ for the C=O stretching vibration, provided supplementary proof that γ-MPS molecules were successfully grafted onto the Mt. For the P-Mt, in addition to these peaks of Mt, the antisymmetric and symmetric stretching vibrations of the –CH_2_ groups were also observed. Moreover, the relative intensities of the absorption bands at 3446.4 and 1639.8 cm^−1^ decreased due to the decrease in the amount of water after the intercalation of TTPC. These results confirmed the successful intercalation of surfactants into the interlayer spacing of Mt, and that the surface had changed from a hydrophilic to a hydrophobic characteristic [31].

For the H-P-Mt, the appearance of strong absorption bands corresponded to the –CH_2_ group, providing supporting information for the successful intercalation of TTPC into the H-Mt. Moreover, the peak intensity weakening of the stretching and bending vibrations of the O–H bands indicated that the amount of hydrated Na^+^ decreased after the ion exchange reaction. The FTIR spectra of the H-Si-Mt not only exhibited the antisymmetric and symmetric stretching bands of the methylene groups, but also had a characteristic band of the C=O stretching vibration. Furthermore, the reduction in the absorption peak intensity of the adsorbed free water and/or hydroxyl groups provided evidence of the successfully intercalated silanes into the interlayer spacing and/or chemically grafted onto the broken edges and surfaces of the H-Mt [32]. A significant increase in the band intensity at 1723.9 cm^−1^ was observed in H-Si-Mt, compared with that of the Si-Mt, indicating that acid activation had a prominent effect on the silylation.

The FTIR spectra of the P-Si-Mt, Si-P-Mt, H-Si-P-Mt, and H-P-Si-Mt, as shown in Figure 2b, provided evidence of the presence of both surfactant and silane molecules. Compared with the P-Si-Mt, as expected, the peak intensity weakening of the methylene groups in the H-P-Si-Mt spectra attested that the acid activation could decrease the amount of ion-exchanged surfactants. Similar weakening was also observed for the H-Si-P-Mt, in contrast with Si-P-Mt. However, it is worth mentioning that the H-P-Si-Mt displayed a greater peak intensity of the C=O band than that of the P-Si-Mt. This result further revealed that a promotion effect was observed on the silylation, due to the acid treatment. Compared with the Si-P-Mt and H-Si-P-Mt, similar results were obtained. In addition, the intensity of the methylene groups was greater for the H-P-Si-Mt relative to the H-Si-P-Mt, which indicated that the surfactant content in the H-P-Si-Mt was higher. Conversely, the silane content in the H-Si-P-Mt was higher than that in H-P-Si-Mt, owing to the appearance of a stronger C=O band. Therefore, it is concluded that the preintercalation or silylation modification of the Mt had a certain hindrance effect on its subsequent modification.

### 3.2. XRD Analysis of Modified Mt

**Figure 3:**
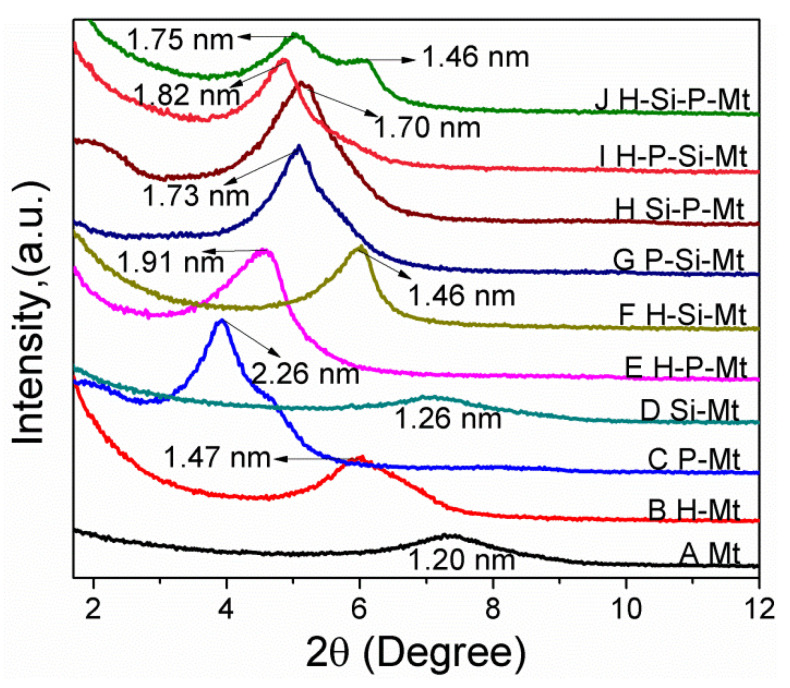
XRD patterns obtained from the Mt and functionalized Mt samples.

The XRD patterns of the Mt and modified Mt are shown in Figure 3. The Mt displayed a reflection at 7.34°, corresponding to a basal spacing of 1.20 nm. This value was in accordance with similar hydrated clays reported by other papers [33,34]. The reflection occurred at 5.97° for the H-Mt, corresponding to a basal spacing of 1.47 nm, which could be attributed to the fact that the hydrochloric acid could remove certain impurities on the Mt surface, open the platelet edges, and consequently increase the surface area [35]. The diffraction peak of P-Mt corresponds to an interlayer spacing of 2.26 nm, indicating that the TTPC surfactants had intercalated and expanded the basal spacing, and a pseudotrimolecular layer orientation of the surfactants was obtained [36]. For the Si-Mt, the reflection peak position shifted slightly to a lower 2θ value, indicating that the γ-MPS had a small effect on the interlayer structure, due to the low level of intercalation. According to the literature [37], the γ-MPS molecules were mainly grafted onto the exterior surfaces and/or the broken edges of the clay. This result also indicated that γ-MPS was not the appropriate molecule for the intercalation to effectively expand the interlayer spacing.

Compared with the H-Mt, the sharp peak (d value = 1.91 nm) of the H-P-Mt clearly showed a bilayer arrangement of TTPC molecules [38]. However, the basal spacing of the H-P-Mt was 0.35 nm smaller than that of the P-Mt, indicating that the amount of intercalated surfactants could be decreased, due to the effective removal of Na^+^. When the H-Mt was further modified by γ-MPS, no significant changes were observed in the XRD peak position, due to the silane molecules being mainly grafted onto the exterior surface and/or at the broken edges. 

The observed small decrease in the basal spacing for the H-P-Mt, after the γ-MPS grafting, decreased from 1.91 nm to 1.82 nm, indicating that the intercalated surfactants were washed out by the hydroalcoholic solution in the process of silylation. However, the basal spacing of the H-P-Si-Mt was bigger than that of the P-Si-Mt, which suggested that a small amount of γ-MPS molecules had been grafted into the interlayer spacing of H-P-Mt. Almost no γ-MPS was grafted into the P-Mt, due to the absence of acid activation pretreatment to improve the amount of hydroxyl groups. For the H-Si-P-Mt, the broadening of the peak was characteristic of a more disordered structure, and two families of modified Mt, with different interlayer spacing (1.75 and 1.46 nm), were formed, indicating the successful intercalation of the surfactants into the H-Si-Mt. The reason was because of the H-Si-Mt being filled with silane molecules, which partly hindered the surfactants ion exchange reaction, resulting in uneven interlayer distances. Compared with the H-Si-P-Mt, the Si-P-Mt presented one basal reflection, and the value of interlayer spacing was similar to the H-Si-P-Mt. It can be concluded that there were almost no silane molecules presented in the interlayer spacing of Si-Mt, and the block effect for the TTPC intercalation was lower than that of H-Si-Mt.

### 3.3. TEM Analysis of Modified Mt

**Figure 4:**
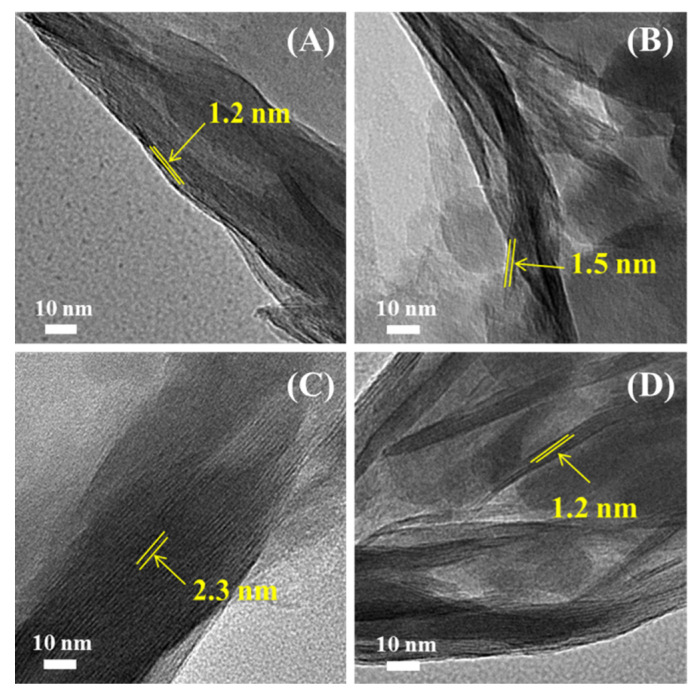
TEM images obtained for (**A**) Mt, (**B**) H-Mt, (**C**) P-Mt and (**D**) Si-Mt samples.

**Figure 5:**
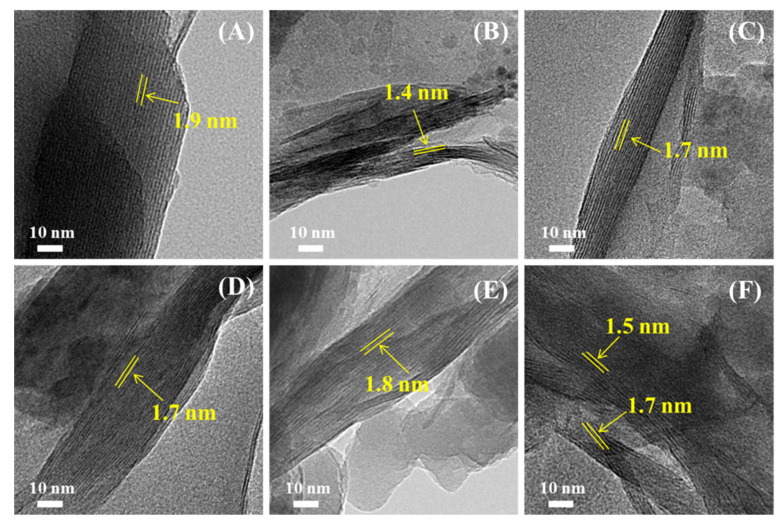
TEM images obtained for (**A**) H-P-Mt, (**B**) H-Si-Mt, (**C**) P-Si-Mt, (**D**) Si-P-Mt, (**E**) H-P-Si-Mt and (**F**) H-Si-P-Mt samples.

To confirm the XRD results, we performed TEM measurements on the investigated Mt samples, as shown in Figure 4 and Figure 5.

The well-ordered multilayered structure was clearly observed for all the investigated samples, before and after modification. The interlayer spacing of the Mt, measured by TEM, was 1.2 nm, which is consistent with the XRD result. The nanolayers of the H-Mt, as shown in Figure 4B, were less densely packed and more expanded, due to the acid activation. Compared with the Mt, the interlayer spacing of the P-Mt was obviously expanded and enlarged, whereas no significant increase was observed for Si-Mt. These results are in good agreement with the XRD data. As for the H-P-Mt and H-Si-Mt (Figure 5A,B), the interlayer spacing, measured by TEM, was 1.9 and 1.4 nm, respectively, which provided significant information about the successful intercalation of the surfactant and the small amount of silane molecules that entered into the H-Mt. The interlayer spacing of the P-Si-Mt, Si-P-Mt, H-P-Si-Mt, and H-Si-P-Mt were all enlarged, and the silicate layers were almost aligned as parallel and ordered lines. Meanwhile, the H-P-Si-Mt and H-Si-P-Mt exhibited analogous TEM images, and the TTPC and γ-MPS were well dispersed, intercalated, or grafted on the samples. Furthermore, the coexistence of the two-layer structure population for the H-Si-P-Mt was also observed. 

### 3.4. BET and BJH Analysis of Modified Mt

The results of nitrogen the adsorption–desorption analysis are stipulated in Table 1. It was observed that the BET surface area and BJH pore volume of Mt obviously increased after acid activation, which was due to the ability of the acid activation to dissolve impurities and open the Mt edges. Similar results were also observed in the comparison of the P-Mt and H-P-Mt, and Si-Mt and H-Si-Mt samples. For the H-P-Mt, it was noticed that the BET surface area and BJH pore volume decreased relative to the H-Mt, which could be explained by the intercalation and filling of the TTPC surfactants. However, an increase in the BET surface area was found in the H-Si-Mt, compared with the H-Mt, due to the grafting of γ-MPS molecules onto the external surfaces and the broken edges. This result once again indicated the successful grafting of γ-MPS molecules onto the external surfaces, rather than into the interlayer spacing.

Compared with the Si-P-Mt, the increase in the BET surface area and BJH pore volume was also observed in the H-Si-P-Mt, due to the acid activation. The decrease in the BET surface area of the H-Si-Mt after modification with the surfactants suggested that the TTPC molecules had been intercalated and filled into the interlayer spacing. On the contrary, compared with the H-P-Mt (80.09 m^2^/g), a larger BET surface area was observed in the H-P-Si-Mt (95.25 m^2^/g). This can be responsible for the hindrance effect of the TTPC surfactants for the γ-MPS molecules, to penetrate into the interlayer spacing. Similar conclusions can also be obtained from the XRD and FTIR results.

### 3.5. XRF Analysis of Modified Mt

Table 2 summarizes the XRF results of the modified samples. A significant reduction in the amount of Na_2_O was observed for the Mt after the acid treatment. The P_2_O_5_ content of the H-P-Mt, and the SiO_2_ content of the H-Si-Mt, significantly increased, respectively, indicating that the surfactant and silane molecules had been introduced into the H-Mt. Moreover, the increase in the P_2_O_5_ percentage in the H-P-Mt suggested that the H-Mt had better interactions with the TTPC molecules, in contrast with the Mt. The percentage of SiO_2_, from 75.3 wt.% for the Si-Mt, increased to 83.1 wt.% for the H-Si-Mt, due to the grafting of more γ-MPS molecules onto the H-Mt. Compared with H-Si-P-Mt, a higher content of P_2_O_5_ and lower content of SiO_2_ were observed in H-P-Si-Mt, indicating that the grafting of the γ-MPS was suppressed, to some extent, by the intercalation of the surfactant. That is to say, the intercalated TTPC molecules will reduce the grafting amount of the γ-MPS. Similarly, the grafted coupling agent will also prevent the further intercalation of the surfactant. This result was also in accordance with the result obtained from FTIR analysis.

### 3.6. TGA-DTG Analysis of Modified Mt

**Figure 6:**
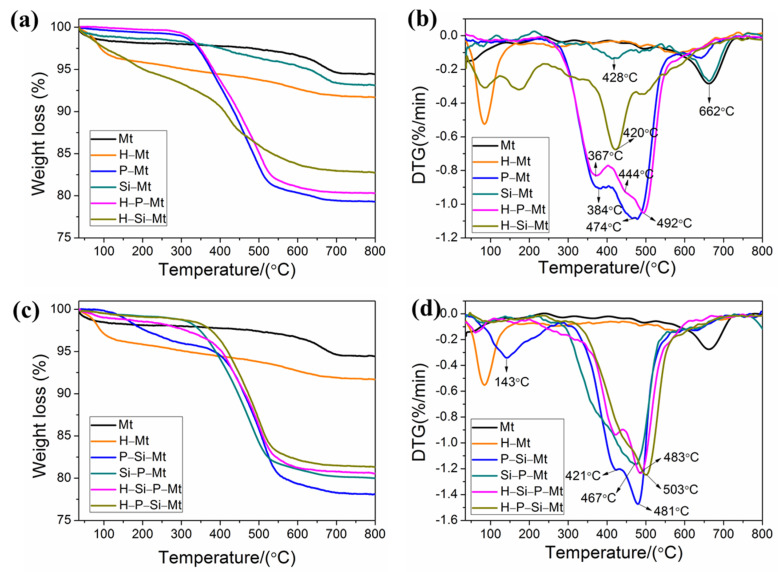
(**a**,**c**) TGA and (**b**,**d**) DTG curves of Mt and functionalized Mt samples.

To gain more insight into the modification process, we investigated the thermal stability of the modified samples, as shown in Figure 6, and the associated results are listed in Table 3. The overall mass loss of the modified Mt was greater than that of the Mt. The following two mass loss stages were obtained for the Mt, as shown in Figure 6a: A mass loss of 1.9 wt.% occurred at temperatures below 200 °C, due to the desorption of the physically adsorbed water. The major mass loss at 662 °C was attributed to the dehydroxylation of the available aluminosilicate groups in the clay layers, and the total mass loss of the Mt was approximately 5.6 wt.%. The Mt was very stable at temperatures of 200–600 °C, which is consistent with previous reports [39]. Similarly, the mass loss of water desorption was observed in the H-Mt, with a significant increase compared with the Mt, indicating the greater hydrophilicity characteristics of the Mt after acid treatment. Beyond the dehydration step, the DTG curves of the H-Mt exhibited one mass loss peak that was related to the dehydroxylation of the OH groups at 602 °C. The mass loss in the H-Mt, below 200 °C, was 4.2 wt.%, and the total mass loss was approximately 8.3 wt.%. However, the P-Mt had a lower mass loss below 200 °C, and a higher mass loss (19.0 wt.%) in the range of 200–600 °C, due to the degradation of the surfactants confined in the interlayer spacing, indicating that the Mt that was intercalated by the TTPC surfactants was much less hydrophilic, which was obviously in favor of the better compatibility with the monomers during the polymerization. Furthermore, the P-Mt had two mass loss peaks that were related to the surfactant decomposition. The fraction of the surfactant that was decomposed at 384 °C was mainly attributed to the surfactants adsorbed by van der Waals forces, and another fraction that had decomposed at 474 °C was associated with the molecules intercalated by ion exchange and adhered by electrostatic interactions [40]. Otherwise, the dehydroxylation mass loss of the layer crystal lattice was substantially lowered for the P-Mt, confirming that the intercalation of the surfactant had truly been achieved. For the Si-Mt, the mass loss (1.4 wt.%) below 200 °C was due to the removal of the physically adsorbed water and methanol solvent. The Si-Mt displayed a peak at 263 °C, which was attributed to the loss of silanes bonded at the broken edges and/or adsorbed on the external surfaces. The peaks at approximately 428 °C and 546 °C were related to the decomposition of the intercalated and chemically grafted silane molecules, respectively [41]. The mass loss of the Si-Mt, in the temperature range of 200–600 °C, was 3.2 wt.%, which was 1.8 wt.% larger than that of Mt, indicating the successful silylation for the Mt layers. However, there was no large increase in the basal spacing after the silylation from the XRD results, which further indicated that the silane was mainly adsorbed and grafted onto the external surfaces and the broken edges. 

For the H-P-Mt, the mass loss of physisorbed water was only 0.2 wt.%, compared with 1.9 wt.% for Mt and 4.2 wt.% for H-Mt, indicating that the H-Mt that was intercalated by the surfactants was much less hydrophilic. In discussing the thermal decomposition of certain modified Mt, the curves, in general, can be divided into four different regions [42]. The first region occurs below 200 °C, and involved the physisorbed water and gases (N_2_ or CO_2_) desorption. The decomposition of the organic species was mainly observed in the interval of 200–600 °C. The dehydroxylation occurred in the range from 600 to 700 °C. Finally, the evolution of products associated with organic carbonaceous residues occurred. The thermal behavior of the organic clays in the second region was very significant, in terms of their compatibility with polymers as nanofillers, because organic substances begin to decompose in this temperature range. The H-P-Mt showed three mass loss peaks in the temperature range of 200–600 °C. The peak at approximately 367 °C was the decomposition of the physically adsorbed surfactants. The peaks at approximately 444 °C and 492 °C were mainly related to the degradation of interlayered adsorbed and intercalated surfactants, respectively [43]. The mass loss of the H-P-Mt in the 200–600 °C temperature range was 18.8 wt.%, which was similar to the P-Mt (19.0 wt.%). However, the interlayer spacing of the P-Mt was 0.41 nm larger than that of the H-P-Mt. Therefore, it could be concluded that the surfactants in the H-P-Mt exhibited a tighter arrangement compared with the P-Mt. Compared with the P-Mt, the maximum mass loss temperature (*T*_max_, obtained from the DTG curve) for the H-P-Mt increased from 474 to 492 °C, which provided additional evidence that the surfactants in the H-P-Mt exhibited a tighter arrangement. The H-Si-Mt showed three distinct thermal events in the temperature range of 200–600 °C that were not observed in the H-Mt, and that corresponded to the decomposition of the silane molecules that were adsorbed onto the surface by hydrogen bonds, intercalation and/or chemical grafting [44]. The peak at 200–350 °C was due to adsorbed silanes on the external surfaces and/or at broken edges, linked by hydrogen bonds to hydroxyl groups. The mass loss between 350 and 450 °C corresponded to the intercalated silanes, whereas that between 450 and 600 °C was due to the grafted molecules on the broken edges and surfaces [45]. In addition, the mass loss of the H-Si-Mt, between 200 and 600 °C, was found to be approximately 11.4 wt.%, which was 8.2 wt.% higher than that of the Si-Mt. This result indicated that a significant increase in the amount of intercalated and/or grafted silane molecules in the H-Si-Mt was obtained, as a result of increasing the available hydroxyl groups during the acid activation. Compared with the Si-Mt, the mass loss of dehydroxylation of the H-Si-Mt was reduced, partly due to the consumption of the hydroxyl groups during the grafting reaction [46]. From the results presented so far, it could be concluded that the acid activation exhibited a beneficial effect on the thermal stability improvement of intercalated surfactants, and the amount of silane molecules grafted onto the surfaces.

From Table 3, the mass loss of H-Si-P-Mt, between 200 and 600 °C, was 17.2 wt.%, which was 5.8 wt.% higher compared to H-Si-Mt and 0.9 wt.% lower compared to Si-P-Mt, indicating that the surfactant had been intercalated into the H-Si-Mt interlayer spacing, but the acid treatment could reduce the amount of intercalated surfactants. This result was in accordance with the XRD results. The two stages of thermal decomposition were observed for the H-Si-P-Mt. The first step, in the range of 200–450 °C, could be contributed to the decomposition of the physically adsorbed TTPC and γ-MPS, as well as the intercalated silane coupling agents, while the second, in the range between 450 and 600 °C, corresponded to the release of the grafted silane and intercalated surfactant [47]. In addition, the *T*_max_ for the H-Si-P-Mt (483 °C) was higher than that for the H-Si-Mt (422 °C) and Si-P-Mt (467 °C). Compared with the H-P-Mt (18.8 wt.%) and P-Si-Mt (18.5 wt.%), the mass loss of H-P-Si-Mt, in the temperature range of 200–600 °C, was 16.9 wt.%, and the decrease was due to the dissolution effect of the hydroalcoholic solution for the intercalated surfactants, and of the hydrochloric acid solution for the exchangeable ions. The H-P-Si-Mt showed two mass loss stages, the same as occurred with the H-Si-P-Mt. However, the *T*_max_ for the H-P-Si-Mt (503 °C) was higher than that for the H-P-Mt (490 °C) and P-Si-Mt (481 °C), confirming that the grafted silane chains, which formed stable siloxane bridges with the clay surface, provided supernumerary thermal stability. According to the above results, it was concluded that the thermal stability of the samples that were modified by three strategies was significantly improved compared with that modified by one or two strategies. Although the organic content of the H-P-Si-Mt was similar to the H-Si-P-Mt, the interlayer spacing of H-P-Si-Mt was larger than that of H-Si-P-Mt. Moreover, the highest result of the *T*_max_ was observed in the H-P-Si-Mt. This result could be reasonably attributed to an increase in the arrangement order of the clay layers for the H-P-Si-Mt.

### 3.7. Thermal Properties of PS/OMt Nanocomposites

**Figure 7:**
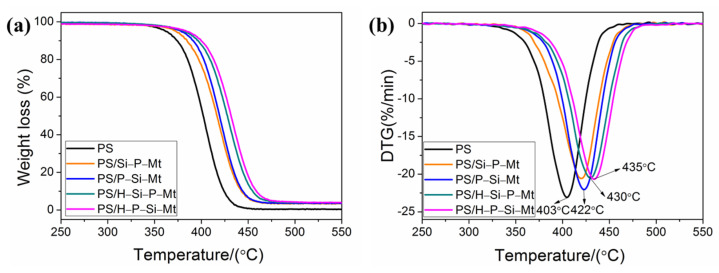
(**a**) TGA and (**b**) DTG curves of pure PS and PS/OMt nanocomposites.

The differences in the TGA-DTG curves of the PS and PS/OMt are shown Figure 7. The samples that were prepared with Si-P-Mt, P-Si-Mt, H-Si-P-Mt, and H-P-Si-Mt were labeled as PS/Si-P-Mt, PS/P-Si-Mt, PS/H-Si-P-Mt, and PS/H-P-Si-Mt, respectively. The degradation temperatures (*T*_-5%_, 5% mass loss temperature; *T*_-50%_, 50% mass loss temperature; *T*_max_, obtained from DTG) are listed in Table 4. 

**Figure 8:**
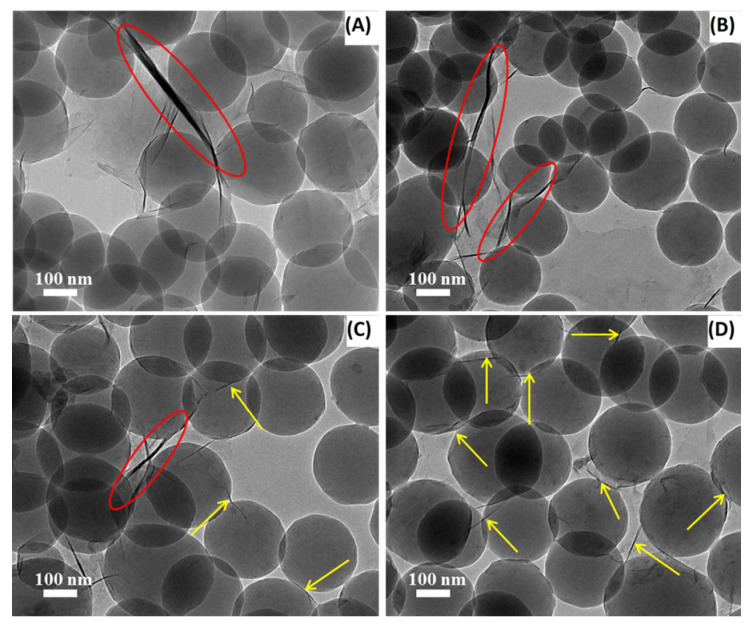
TEM images obtained for (**A**) PS/Si-P-Mt, (**B**)PS/P-Si-Mt, (**C**) PS/H-Si-P-Mt and (**D**) PS/H-P-Si-Mt nanocomposites.

It is worth mentioning that the thermal stability of the PS/OMt nanocomposites was considerably enhanced compared with pure PS, which could be associated to the nanoscale OMt layers, with a large surface preventing the out-diffusion of the volatile decomposition products. At 5% mass loss, the *T*_−5%_ of PS/Si-P-Mt, PS/P-Si-Mt, PS/H-Si-P-Mt, and PS/H-P-Si-Mt were about 374 °C, 379 °C, 382 °C, and 386 °C, respectively, which was 15 °C, 20 °C, 23 °C, and 27 °C higher than that of PS. Also, the *T*_max_ was improved from 403 °C for the PS, to 419 °C, 422 °C, 430 °C, and 435 °C for PS/Si-P-Mt, PS/P-Si-Mt, PS/H-Si-P-Mt, and PS/H-P-Si-Mt, respectively. These indicated that the P-Si-Mt exhibited a better effect on the thermal stability improvement of PS than the Si-P-Mt. Additionally, the PS/H-Si-P-Mt and PS/H-P-Si-Mt had relatively higher decomposition temperatures as compared with those of the PS/Si-P-Mt and PS/P-Si-Mt. This could be attributed to a better interfacial interaction and dispersion that was formed in the polymer matrix for the triple-modified nanofillers. In contrast to the double-functionalized nanofillers, the triple-modified clays tend to create a better labyrinth pathway, to slow down the diffusion of volatile gas in the polymer matrix, as illustrated in Figure 8. According to previous studies [47,48], a better improvement in thermal stability for the nanocomposites with different fillers was observed in air or oxygen, compared to a nitrogen atmosphere. Under an oxidizing atmosphere, the differences in thermal stability between PS and PS/OMt will be, very likely, much higher, due to the shielding effect of OMt on the diffusion of gases in the polymer. As denoted by the red circles, the tightly stacked silicate region in the TEM images of Figure 8A,B clearly showed a typical intercalated structure, with narrow interlayer spacing. However, in the case of the H-Si-P-Mt sample, the nanocomposites showed a mixture of both intercalated and exfoliated structures, revealing an intermediate state from intercalation to exfoliation. The exfoliated structures, which indicated that the individual silicate layers were kept free from the adjacent clay layers in the polymer matrix, were observed in the Figure 8D, as denoted by the yellow arrows. These proved that the triple-functionalized samples possessed greater affinity and better interaction towards the PS matrices, due to the larger interlayer spacing, relative to the dual-functionalized samples. Our group has previously shown that the polystyrene chains can enter into the OMt to increase the interlayer spacing of OMt, leading to intercalated and/or exfoliated structures in the resulting nanocomposites, during the soap-free emulsion polymerization [25]. The complete exfoliation of the H-P-Si-Mt nanofiller in the PS matrix was also due to a larger space for the ease of the PS chains penetration. Meanwhile, the presence of a higher content of γ-MPS with the C=C groups acted as the crosslinking agent, connecting the silicate and PS chains, which was covalently bonded on the Mt surface to increase the thermal properties of the nanocomposites [49]. With higher thermal stability, the PS/H-P-Si-Mt nanocomposite particles showed promising applications in the lubricating materials for oil and gas drilling engineering.

The best improvements in the thermal stability of polystyrene nanocomposites, under a nitrogen atmosphere, obtained in this work and in other works from the literature, using this and other types of fillers, are summarized in Table 5. The better improvement of the thermal stability of polystyrene nanocomposites was observed in this work, using very small amounts (3 wt.% loadings) of these fillers.

## 4. Conclusions

This investigation has demonstrated a novel approach to determine the proper strategy for synthesizing high thermally resistant modified Mt, with enlarged interlayer spacing. The effects of various strategies combining acid activation, ion exchange, and silylation, on the modification of Mt, have been studied. After the acid treatments, increases in the BET surface area and BJH pore volume, and reductions in the amount of Na^+^, were observed for the sample. The amount of chemical grafting of the γ-MPS was increased with the acid treatment, whereas the amount of intercalation of the TTPC surfactant was decreased. According to the XRD and TEM analysis, a significant interlayer spacing enhancement was observed in the TTPC surfactant-treated samples. On the contrary, no significant changes were obtained when the silylation reaction was performed on the samples. The results of FTIR and XRF revealed that the preintercalation or silylation modification of the Mt had a certain hindrance effect on its subsequent modification. The higher thermally resistant modified OMt that was found for samples that were treated by three different treatments, indicates that both the samples have the potential to act as effective nanofillers in the polymer matrix. The PS nanocomposites using triple-modified Mt showed much higher thermally resistant stability than pure PS and PS nanocomposites reinforced by double-modified samples. The *T*_-5%_ and *T*_max_ of the PS nanocomposites using H-P-Si-Mt were noticeably enhanced to 386 °C and 435 °C (increased by 27 °C and 32 °C), respectively, compared with the neat PS. This was mainly attributed to the exfoliated two-dimensional layers with large surface areas, which increased the dispersion and interfacial interaction in the polymer matrix, resulting in the formation of a more complex tortuous pathway, to constraint the diffusion of volatile decomposition products. This approach of modification for Mt opened opportunities for the design of polymer/clay nanocomposites with enhanced thermal properties, which was an interesting result for applications in lubricating materials in oil and gas drilling engineering.

## Figures and Tables

**Table 1:** Results of nitrogen adsorption–desorption analysis.

Sample	BET Surface Area (m^2^/g)	BJH Pore Volume (cm^3^/g)
Mt	40.36	0.173
H-Mt	105.93	0.259
P-Mt	30.12	0.163
Si-Mt	68.87	0.191
H-P-Mt	80.09	0.244
H-Si-Mt	108.29	0.260
P-Si-Mt	33.90	0.168
Si-P-Mt	31.62	0.154
H-Si-P-Mt	56.58	0.212
H-P-Si-Mt	95.25	0.238

**Table 2:** Distribution of the composition of Mt and functionalized Mt samples found by XRF measurement.

Sample	Composition (wt.%)									
	SiO_2_	Al_2_O_3_	MgO	Na_2_O	CaO	Fe_2_O_3_	K_2_O	P_2_O_5_	TiO_2_	Others ^a^
Mt	72.9	14.7	4.1	3.2	1.5	1.3	0.3	0	0.1	1.9
H-Mt	81.0	13.2	2.7	0.4	0.2	1.3	0.3	0	0.1	0.8
P-Mt	71.6	14.5	4.1	1.5	1.5	1.5	0.3	2.9	0.1	2.0
Si-Mt	75.3	13.6	3.8	2.5	1.3	1.0	0.3	0	0.1	2.1
H-P-Mt	77.7	13.2	2.8	0.1	0.2	1.2	0.3	3.7	0.1	0.7
H-Si-Mt	83.1	12.0	2.5	0.3	0.2	1.3	0.3	0	0.1	0.2
P-Si-Mt	72.8	14.3	4.0	0.9	1.6	1.6	0.3	2.7	0.1	1.7
Si-P-Mt	71.5	14.5	4.0	1.1	1.5	1.5	0.3	3.4	0.1	2.1
H-Si-P-Mt	81.4	12.3	2.4	0.1	0.1	1.1	0.3	1.8	0.1	0.4
H-P-Si-Mt	78.7	13.3	2.7	0.1	0.2	1.3	0.3	2.5	0.1	0.8

^a^ Others = % SO_3_ + % F + % Cl.

**Table 3:** Relative mass loss during decomposition of Mt and modified Mt samples (wt.%).

Sample	Temperature Range		
	<200 °C	200–600 °C	30–800 °C
Mt	1.9	1.4	5.6
H-Mt	4.2	2.8	8.3
P-Mt	0.6	19.0	20.7
Si-Mt	1.4	3.2	6.8
H-P-Mt	0.2	18.8	19.6
H-Si-Mt	5.0	11.4	17.2
P-Si-Mt	2.4	18.5	21.9
Si-P-Mt	0.9	18.1	19.9
H-Si-P-Mt	1.5	17.2	19.4
H-P-Si-Mt	0.8	16.9	18.6

**Table 4:** TGA and DTG data of pure PS and PS/OMt nanocomposites.

Sample	*T*_−5%_ (°C)	*T*_−50%_ (°C)	*T*_max_ (°C)
PS	359	401	403
PS/Si-P-Mt	374	416	419
PS/P-Si-Mt	379	420	422
PS/H-Si-P-Mt	382	427	430
PS/H-P-Si-Mt	386	432	435

**Table 5:** Parameters of enhanced thermal properties.

Filler	Method	Content (wt.%)	Δ*T_onset_* (°C)	Δ*T*_−50__%_ (°C)	Δ*T_max_* (°C)	Ref.
Organoclay	Bulk polymerization	5		50		[50]
Organoclay	Bulk polymerization	3	50			[51]
Organoclay	Solvent blending	5		16	24	[52]
Organoclay	Solution polymerization	5	51.1			[53]
Organoclay	In situ intercalative polymerization	10	109.5			[54]
Fullerene	Melt extrusion	1	7.0	4.5	2.2	[47]
Layered double hydroxide	Soap-free emulsion polymerization	1	53.3			[55]
ZnAl-layered double hydroxide	Solution intercalation	10		39		[56]
Cadmium sulfide	Melt extrusion	10			95	[57]
Aluminum hypophosphite	Melt extrusion	30	–23		6	[58]
Calcium carbonate	Solution casting	4			15	[59]
Organoclay	Soap-free emulsion polymerization	3	27	31	32	This work

Δ: the corresponding variations in thermal stability of each composite compared with the pure polymer.

## Data Availability

Not applicable.
